# Amplification of the *CD24* Gene Is an Independent Predictor for Poor Prognosis of Breast Cancer

**DOI:** 10.3389/fgene.2019.00560

**Published:** 2019-06-12

**Authors:** Peng Zhang, Pan Zheng, Yang Liu

**Affiliations:** ^1^Division of Immunotherapy, Institute of Human Virology, University of Maryland School of Medicine, Baltimore, MD, United States; ^2^OncoImmune, Inc., Rockville, MD, United States

**Keywords:** breast cancer, biomarker, copy number variation, *CD24*, *TP53*, basal-like

## Abstract

*CD24* is a glycosyl-phosphatidyl-inositol linked glycoprotein expressed in a broad range of cell types including cancer cells. Although it is overexpressed in nearly 70% of human cancers, copy number variation of the *CD24* locus has not been reported for any cancer. Here, we analyzed the genomics, transcriptomics, and clinical data of 1082 breast cancer (BRCA) samples and other cancer samples from the clinically annotated genomic database, The Cancer Genome Atlas (TCGA). The GISTIC2 method was applied to stratify the *CD24* copy number, and Cox regression was performed to compare hazard ratio (HR) of *CD24* overexpression, amplification and other traditional prognosis features for overall survival (OS). Our data demonstrated that *CD24* amplification strongly correlated with its mRNA overexpression as well as *TP53* mutant, cancer proliferation and metastasis features. In particular, *CD24* amplification was enriched in basal-like subtype samples and associated with poor clinical outcome. Surprisingly, based on the univariate Cox regression analysis, *CD24* overexpression (*HR* = 1.62, *P* = 0.010) and copy number amplification (*HR* = 1.79, *P* = 0.022) was more relevant to OS than *TP53* mutant, mutation counts, diagnosis age, and BRCA subtypes. And based on multivariate survival analysis, *CD24* amplification remained the most significant and independent predictor for worse OS (*HR* = 1.88, *P* = 0.015).

## Introduction

*CD24* is located on chromosome 6q21 and encodes a glycosylphosphatidylinositol-linked cell surface glycoprotein (Hough et al., 1994). It is expressed on hematopoietic cells (Li et al., 2004; Israel et al., 2005), neural cells (Nielsen and Cohen, 1996), epithelial cells (Sleeman et al., 2006), muscle cells (Higuchi et al., 1999), stem cells (Lawson et al., 2006; Shackleton et al., 2006), and many other cell types including cancer cells (Kristiansen et al., 2002; Fillmore and Kuperwasser, 2007; Sagiv et al., 2008). In addition to the immunological functions (Li et al., 2004, 2006; Liu and Zheng, 2007), recent studies have implicated *CD24* function in tumorigenesis and progression of multiple cancer types, including carcinomas in lung, prostate, ovarian, breast, and brain (Kristiansen et al., 2002, 2003; Fillmore and Kuperwasser, 2007; Sagiv et al., 2008). Cell surface *CD24* has been shown to contribute to tumor metastasis and *Src* oncogene activation. More recently, we and others have reported that *CD24* is translocated to nuclei, where it affects the stability of tumor suppressor gene *P14ARF* and *TP53* (Wang et al., 2015). In particular, we have found that *CD24* silencing could prevent functional inactivation of p53 by both somatic mutation and viral oncogenes, and that *TP53* mutated at a higher rate among glioma and prostate cancer samples with higher *CD24* mRNA levels (Wang et al., 2015). As a result, several tumor cell lines have been shown to be oncogenic addicted to *CD24* overexpression as their growth and metastasis are diminished upon inactivation of *CD24* expression. However, despite the well-documented function of *CD24* overexpression in tumorigenesis, the driving force of *CD24* overexpression in cancer has not been systematically investigated.

Tumorigenesis is driven by a combination of inherited and acquired genetic alterations. Many studies, including reports from TCGA project, have made use of multiplatform genomic analysis to identify known and new genetic drivers of tumor phenotypes (Hodis et al., 2012; Chen et al., 2016). Copy number variation refers to either extra or missing copies of a gene. Gene copy number amplification is a major genetic mechanism to increase the expression of oncogenes. For example, amplification of *ERBB2*, the gene encoding human epidermal growth factor receptor two, has been reported in approximately 20% of BRCAs and used for therapeutic decision (Bartlett et al., 2001). Likewise, the *MYC* oncogene amplification has been established in numerous cancer types and has emerged as a defining feature for the classification of medulloblastoma (Ramaswamy et al., 2016). Therefore, it is of considerable interest to determine whether *CD24* is amplified in human cancers, and if so whether such amplification corresponds to *CD24* overexpression and clinical outcome. Here, we investigated the copy number status and expression level of *CD24* in BRCA, ovarian cancer, lung cancer, and prostate cancer. We reported *CD24* amplification in carcinoma of breast, ovarian, lung but not in the prostate, and the copy number amplification was strongly correlated with *CD24* mRNA overexpression, which in turn correlated with signature genes of tumor growth and metastasis. Most importantly, *CD24* gene amplification seemed to be the most impactful genetic alteration for the prognosis of BRCA.

## Materials and Methods

### Datasets

**FIGURE 1:**
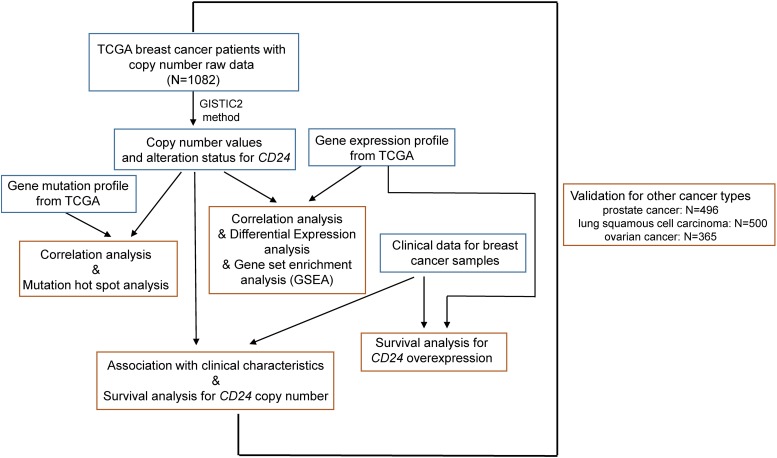
Illustration of study workflow. The flowchart of data collection and method implementation in this work.

We collected the largest publicly available cancer genomics database namely TCGA with genomic, transcriptomic, and clinical data (Figure 1). We accessed the TCGA data portal^1^ and downloaded mRNA expression quantification profiles (HTSeq–FPKM) and masked copy number segment profiles for BRCA (*N* = 1082), prostate cancer (*N* = 496), lung squamous cell carcinoma (*N* = 500), and ovarian cancer (*N* = 365). Clinical data files and annotated mutation files of cancer samples were downloaded from cBioPortal for Cancer Genomics^2^.

### Copy Number Analysis

GISTIC2 (Mermel et al., 2011) method was applied to the transformed copy number segment data, with a noise threshold used to determine copy gain or loss. We performed GISTIC version 2.0.22 by using the Homo sapiens (hg38) RefSeq gene annotations^3^. The copy number values were obtained by examining the distribution of log2 ratios to identify peaks associated with copy number states. The default GISTIC threshold for identifying gains and losses (0.1 and −0.1, respectively) were used. Other GISTIC parameters were the following: genegistic = 1, maxseg = 2,000, js = 2, cap = 1.5, broad = 1, brlen = 0.7, conf = 0.98, armpeel = 1, rx = 0 and gcm = extreme. The GISTIC algorithm takes into account both high and low thresholds for copy number determination across all the input samples to assign significance to copy number variation. The copy number status of low-level gene amplification, high-level gene amplification, low-level gene deletion, and high-level gene deletion was inferred using the “thresholded” calls.

### Gene-Set Enrichment Analysis

Gene-set enrichment analysis was performed with the GSEA program (v. 3.0) (Subramanian et al., 2005). The Broad Molecular Signatures Database (MSigDB v6.0) set H (hallmark gene sets) was used, which summarize and represent specific well-defined biological states or processes. The GSEA program was run with 1,000 permutations for statistical significance estimation, and the default signal-to-noise metric between the two phenotypes was used to rank all genes.

### Survival Analysis

Univariate and multivariate survival analysis was performed by using the Cox proportional hazard regression model with OS time (5-year) to assess the prognostic value of gene expression, copy number variation, and clinical characteristics (Figure 1). The prognostic value of discrete variables was estimated by Kaplan–Meier survival curves, and the log-rank test was employed to estimate the significance among different survival curves.

### Biostatistical Analysis

Data were analyzed by using an unpaired Mann-Whitney test to compare between two groups and one-way analysis of variance (ANOVA) for multiple comparisons. Fisher’s exact test was used for enrichment analysis. The Spearman correlation coefficient was performed to estimate the strength and significance of the association between two continuous variables, such as putative copy number values and mRNA expression (Figure 1). For the differential expression analysis, the Mann-Whitney test with multiple testing adjustment (False Discovery Rate, FDR) determined the significant difference. In the graphs, *y*-axis error bars represent median with 95% CI as indicated. Statistical calculations were performed using GraphPad Prism software (GraphPad Software, San Diego, CA) or R Software^4^.

## Results

### *CD24* Overexpression Predicts Adverse Prognosis in BRCA Patients

**FIGURE 2:**
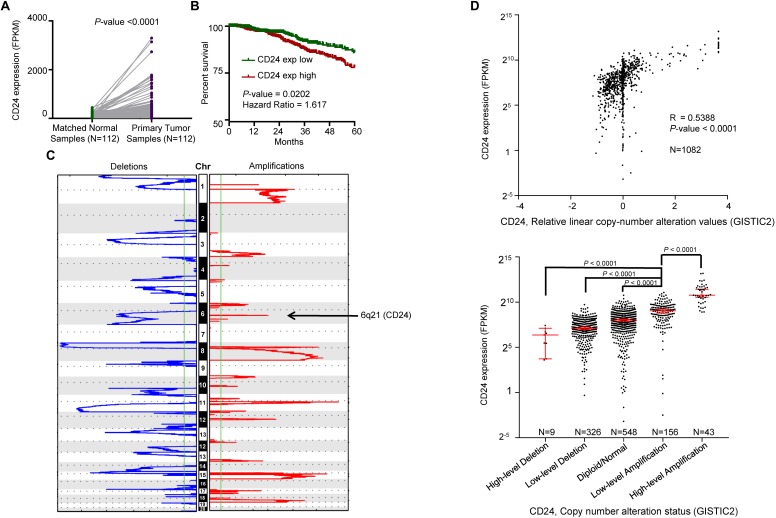
Correlation between *CD24* copy number alterations and mRNA expression. **(A)** Change of *CD24* mRNA expression between tumor samples and matched normal samples from TCGA BRCA studies (*N* = 112). **(B)** Kaplan–Meier survival curve comparing the high (*N* = 533) and low (*N* = 532) expression value of *CD24* (determined by the median value) for the TCGA BRCA patient cohort. **(C)** GISTIC heat map showing genomic copy-number profiles from TCGA BRCA studies. Gain (red) and loss (blue) of each peak are shown. Regions of copy-number gain (6q21) were highlighted. **(D)** Scatter plot (top) and dot plot (bottom) showing the strong positive correlation between *CD24* copy number values defined by GISTIC2 approach and mRNA expression values quantified by FPKM (*N* = 1082). GISTIC2 method stratified the *CD24* copy number values into five categories: high-level copy number deletion (*N* = 9), low-level copy number deletion (*N* = 326), diploid normal copy (*N* = 548), low-level copy number amplification (*N* = 156), and high-level copy number amplification (*N* = 43). Statistical significance was determined by the Paired *t*-test in panel **(A)**, log-rank test in panel **(B)** and the One-way ANOVA in panel **(D)** (bottom). The Spearman coefficient of correlation (R) and significance in panel **(D)** (top) was determined by linear regression.

We first determined the mRNA expression of *CD24* in BRCA samples from TCGA, which revealed the transcripts of *CD24* were dramatically enhanced in paired BRCA samples (*N* = 112) compared with adjacent breast normal tissues (*P*° < °0.0001) (Figure 2A). The upregulation was also observed when unpaired tumors were compared with normal samples (*P*° < °0.0001) (Supplementary Figure S1A). Since *CD24* is abundantly expressed in hematopoietic cells, we evaluated whether *CD24* transcripts were affected by the extent of leukocyte infiltration using the common leukocyte antigen *CD45* as a reference. We observed no difference in *CD45* transcripts between *CD24*^high^ and *CD24*^low^ BRCA samples (Supplementary Figure S1B). Therefore, the difference in *CD24* transcript levels among BRCA samples was not due to a difference in leukocyte infiltration. Then, we investigated the clinical implication of *CD24* overexpression in BRCA patients. Kaplan–Meier survival analysis revealed that higher *CD24* mRNA expression significantly associated with worse OS in patients with BRCA (*N* = 1079, *P* = 0.0102; Figure 2B). And our *in silico* analysis of the other two independent BRCA cohorts (Chanrion et al., 2008; Clarke et al., 2013) confirmed this correlation (Supplementary Figure S1C). Besides, higher *CD24* expression also predicted poor metastasis-free survival for patients in BRCA metastasis patient cohorts (Desmedt et al., 2007; Minn et al., 2007), as evidenced by Kaplan–Meier curves shown in Supplementary Figure S1D. In summary, *CD24* is significantly upregulated in BRCA, and its overexpression is an adverse prognostic factor for BRCA patients.

### Copy Number Amplification Correlates to *CD24* Up-Regulation in BRCA

We interrogated the chromosomal segment value of TCGA BRCA dataset for significant copy number alterations. As expected, our analysis confirmed many known copy number amplifications, including those observed in 3q26.1 (*PIK3CA*) (Wu et al., 2005), 8q24.21 (*MYC*) (Rodriguez-Pinilla et al., 2007), and 11q13 (*CNCD1*, *EMS1*) (Ormandy et al., 2003). We also observed the chromosomal region of 6q21 (encompassing *CD24*) harbored a major amplification (Figure 2C). And a significant correlation between *CD24* copy number values and mRNA expression was found among all BRCA samples (Figure 2D, *R* = 0.5388, *P*-value <0.0001). Moreover, we stratified BRCA patients into five groups (High-level Deletion, *N* = 9; Low-level Deletion, *N* = 326; Diploid, *N* = 548; Low-level Amplification, *N* = 156; High-level Amplification, *N* = 43) based on *CD24* copy number values by using GISTIC2 framework. As shown in Figure 2D, approximately one-fifth of all BRCA samples harbored *CD24* amplification, which was on par with *ERBB2* amplification. Consistently, BRCA samples harboring *CD24* amplification exhibited highest mRNA expression than those that exhibit diploid *CD24*, while *CD24* deletion samples had the lowest mRNA expression of *CD24* (Figure 2D). Therefore, the gain of copy number is probably a major mechanism that contributes to the up-regulation of *CD24* in BRCA.

### *CD24* Amplification Positively Correlated With Cell Proliferation and *MYC* Signaling Pathway

**FIGURE 3:**
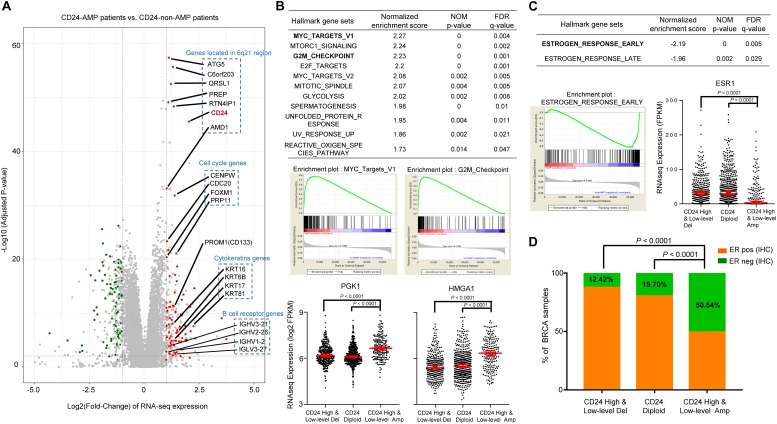
*CD24* amplification correlated with cell cycle and estrogen response activity. **(A)** Volcano plot of mRNA expressions change between BRCA samples harboring *CD24*-amplification and non-*CD24*-amplification. The *x*-axis specifies the fold-changes (FC) and the *y*-axis specifies the negative logarithm to the base 10 of the adjusted *p*-values. Gray vertical and horizontal dashed lines reflect the filtering criteria. Red and green dots represent genes expressed at significantly higher (*n* = °79) or lower (*n* = °68) levels, respectively. The genes located in the 6q21 chromosomal region and enriched in functional patterns are highlighted. **(B)** GSEA comparing gene-expression signatures of TCGA breast tumors with the *CD24*-amplification and non-*CD24*-amplification. GSEA positive result table (top) showing significant enrichment (*FDR* < 0.05) of the hallmark gene sets from MSigDB. The enrichment plot on the middle left (right, respectively) shows the distribution of MYC targets (G2M checkpoint, respectively) genes that are positively correlated with the *CD24*-amplification phenotype. The distribution plot on the bottom shows the marker genes (*PGK1* and *HMGA1*) expression difference among BRCA patients with different *CD24* copy number status. **(C)** GSEA negative association table (top) showing significant enrichment (*FDR* < 0.05) of the hallmark gene sets from MSigDB. The enrichment plot on the bottom left shows the distribution of estrogen response early genes that are negatively correlated with the *CD24*-amplification phenotype. The distribution plot on the bottom right shows the marker genes (*ESR1*) expression difference among BRCA patients with different *CD24* copy number status. **(D)** Distribution of ER-negative (based on IHC) samples among different *CD24* copy number groups shows the patients with *CD24*-amplification are enriched in ER-negative status. Normalized enrichment score (NES) and FDR *q*-value of GSEA are shown in panels **(B,C)**. Statistical significance was determined by the One-way ANOVA in panels **(B,C)**, and the exact Fisher’s test in panel **(D)**.

To gain insights into the molecular mechanisms underlying pro-tumorigenic action of *CD24* amplification in BRCA cells, we first analyzed differential gene expression patterns between *CD24* amplified samples (*N* = 199) and *CD24* non-amplified samples (*N* = 883) in TCGA BRCA dataset (Figure 3A). In total, 79 genes were identified as significantly up-regulated genes, which include six genes (*ATG5, C6orf203, QRSL1, PREP, RTN4IP1*, and *AMD1*) located very close to *CD24* genomic region at chromosomal loci. More interesting, we found four cell cycle genes (*CENPW, CDC20, FOXM1*, and *PRP11*) and four cytokeratin genes (*KRT16, KRT6B, KRT17*, and *KRT81*) of these up-regulated expressed genes, which indicating that multiple genes involved in cancer cell proliferation and invasion pathway may be activated concordantly. Besides, several B cell receptor genes were enriched among *CD24*^hi^ BRCA, the significance of such enrichment remains to be elucidated.

We further performed gene set enrichment analysis (GSEA) using the MSigDB hallmark gene sets (Subramanian et al., 2005), which revealed that a large number of gene sets were positively enriched in samples harboring *CD24* amplification compared with *CD24* non-amplification samples (Figure 3B). Among the 11 significantly enriched gene sets, the groups of “*MYC* targets” (including V1 and V2), whose expression is connected to c-*Myc*-dependent phenotypes such as cellular proliferation, transformation, or apoptosis, showed particularly strong enrichment, which was confirmed by dramatically up-regulated expression value of *PGK1* (Tang et al., 2009), a *MYC* target marker gene, in *CD24* amplification samples (*P* < 0.0001; Figure 3B). Notably, genes involved in the “G2/M checkpoint” (genes involved in progression through the cell division cycle) and “*E2F* targets” (genes encoding cell cycle related targets of *E2F* transcription factors) were also highly positively enriched in patients harboring *CD24* amplification. As an example, *HMGA1*, an *E2F* target gene that marks the G2M checkpoint (Schuldenfrei et al., 2011), was significant up-regulated *CD24* amplification samples (*P* < 0.0001; Figure 3B). In summary, these data demonstrate a positive correlation between *CD24* amplification and cell proliferation, a key step in oncogenesis.

### *CD24* Amplification Negatively Correlates With Estrogen Response Activity in BRCA

Gene set enrichment analysis showed that only two hallmark gene sets (“estrogen response early” and “estrogen response late”) were negatively correlated with *CD24* amplification (Figure 3C). Then we examined the mRNA expression of human ER (*ESR1*) among BRCA patients with different *CD24* copy number status. As shown in Figure 3C, *ESR1* decreased dramatically in BRCA patients harboring *CD24* amplification (*P* < 0.0001). We further accessed the ER status based on IHC data of TCGA BRCA patients and compared the ER-negative rates among different *CD24* copy number groups. We observed a significantly higher rate of ER-negative patients in *CD24* amplification groups compared with *CD24* diploid and deletion groups (Figure 3D, *P* < 0.0001).

### *CD24* Amplified Samples Exhibit a Selective Increase of *TP53* Mutations

**FIGURE 4:**
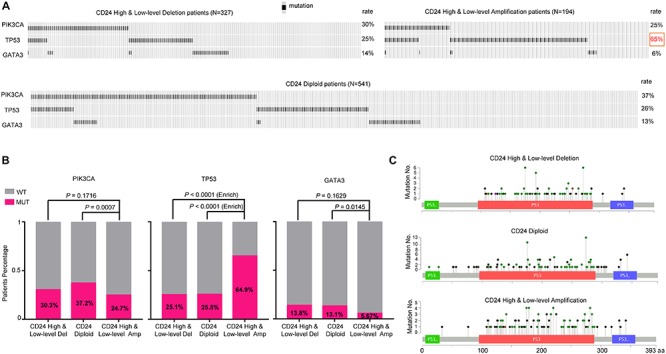
*TP53* mutation is selectively enriched in *CD24* amplification samples. **(A)** cBioPortal OncoPrint plot showing the distribution of *PIK3CA*, *TP53* and *GATA3* mutation rate in the TCGA BRCA dataset. **(B)** Bar graphs showing the percentage of TCGA BRCA samples with mutations in *PIK3CA, TP53*, and *GATA3* by different *CD24* copy number groups. **(C)** cBioPortal Lollipop plot showing the loci distribution of mutations in *TP53* across TCGA BRCA samples.

To determine whether *CD24* amplification tumors were enriched for the mutations of driver genes, we evaluated its association with the mutation profile of three most commonly altered genes (*PIK3CA, TP53*, and *GATA3*) in BRCA. We used the OncoPrint function of cBioPortal for Cancer Genomics tools^5^ to explore the mutation rate of these three genes in TCGA BRCA dataset (Figure 4A). Remarkably, the mutation rate of *TP53* in *CD24* amplified samples is 2.6-fold higher than the non-amplified samples, reaching nearly 65% of the total samples (Figure 4B). In contrast, the *CD24* amplified samples have reduced mutation rate of *PIK3CA* and *GATA3* genes. Nevertheless, there was no hotspot mutation of *TP53* that was specifically enriched in any groups (Figure 4C). These data are consistent with our previous report showing a critical role for *CD24* in inactivation of mutant p53 proteins (Wang et al., 2015).

### Prognostic Significance of *CD24* Amplification in BRCA

**FIGURE 5:**
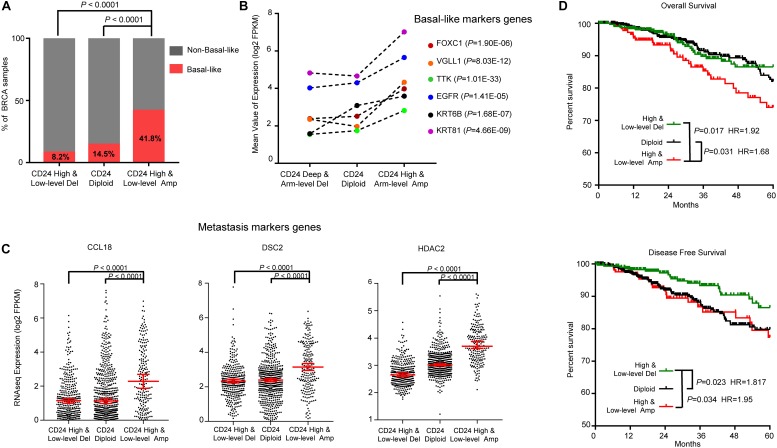
*CD24* amplification associated with basal-like subtype and poorer survival of BRCA patients. **(A)** Distribution of Basal-like samples among different *CD24* copy number status subtypes of BRCA from TCGA patient cohort. **(B)** Mean value of Basal-like marker genes in BRCA. **(C)** The distribution plot of breast metastasis marker genes expression among BRCA patients with different *CD24* copy number status. **(D)** Kaplan–Meier survival curves comparing the OS and disease-free survival time for different *CD24* copy number subtypes in BRCA. Statistical significance was determined by the exact Fisher’s test in panel **(A)**, Wilcoxon test in panel **(B)**, One-way ANOVA in panel **(C)**, and log-rank test in panel **(D)**.

Breast cancer is a heterogeneous disease with diverse pathological features and survival outcomes (Sorlie et al., 2003), and the basal-like or triple-negative BRCAs (TNBCs, lacking expression of the ER, PR and HER2), characterized by a poor prognosis and no specific targeted therapies group (Badve et al., 2011). We first investigated the distribution of basal-like patients among different *CD24* copy number groups. As shown in Figure 5A, Basal-like patients were significantly enriched in *CD24* amplification groups compared with *CD24* diploid and deletion groups (both *P* < 0.0001), which was confirmed by dramatically up-regulated expression of six basal-like genes (*FOXC1* (Ray et al., 2010), *VGLL1* (Castilla et al., 2014), *TTK* (Rakha et al., 2008), *EGFR* (Cheang et al., 2008), *KRT6B* (Lehmann et al., 2011), *KRT81* (Lehmann et al., 2011); Figure 5B).

We further examined the distribution of three BRCA metastasis marker genes (*CCL18* (Chen et al., 2011), *DSC2* (Landemaine et al., 2008) and *HDAC2* (Roy et al., 2014); Figure 5C) expression values. Consistent with the correlation between *CD24* amplification and BRCA metastasis (Supplementary Figure S1D), the marker genes of BRCA metastasis are significantly up-regulated in *CD24* amplification groups.

To determine if the copy number amplification of *CD24* has prognostic value on the clinical outcome of BRCA patients, we employed a Kaplan–Meier survival analysis for TCGA BRCA patient cohort. As shown in Figure 5D, BRCA patients with the *CD24* amplification had significantly poorer survival rates compared with the *CD24* diploid (*P* = 0.031) and *CD24* deletion (*P* = 0.017) patients. In addition, *CD24 deletion* increased the probability of disease-free survival (Figure 5D).

To further assess the prognostic potential of *CD24* amplification in BRCA, we performed the univariate and multivariate Cox regression analysis, including diagnosis age, tumor subtype, mutation count, *TP53* mutant status and *CD24* expression value. The multivariate result adjusted for standard clinical and pathological parameters confirmed that the impact of *CD24* amplification on OS was independent of BRCA subtype and *TP53* mutant status (Table 1). Altogether, the results show that *CD24* amplification is a biomarker to predict both clinical Basal-like stratification and adverse outcome for BRCA patients.

**TABLE 1:** Cox regression analysis in TCGA BRCA patients.

**Characteristics**	**Univariate**		**Multivariable**	
	**Hazard ratio (95% CI)**	***P*-value**	**Hazard ratio (95% CI)**	***P*-value**
CD24_CNV (Amp vs. Non-Amp)	1.79 (1.15–2.79)	0.010	1.88 (1.13–3.12)	0.015
CD24_Expression (High vs. Low)	1.62 (1.07–2.44)	0.022	1.32 (0.83–2.10)	0.243
CD45_Expression (High vs. Low)	0.66 (0.44–0.98)	0.041	0.69 (0.44–1.06)	0.093
TP53_Mutation (Mutated vs. WT)	1.35 (0.90–2.03)	0.147		
Patient mutation count	1.00 (1.00–1.00)	0.005	1.00 (1.00–1.00)	0.005
Diagnosis age	1.04 (1.02–1.05)	2*e*-06	1.03 (1.02–1.06)	1.78*e*-05
**Subtype (vs. Luminal)**				
Basal-like	1.38 (0.80–2.39)	0.252		
HER2-enriched	2.36 (1.26–4.44)	0.007		
Normal-like	2.04 (0.87–4.76)	0.099		

### *CD24* Amplification in Prostate, Lung and Ovarian Cancers

**FIGURE 6:**
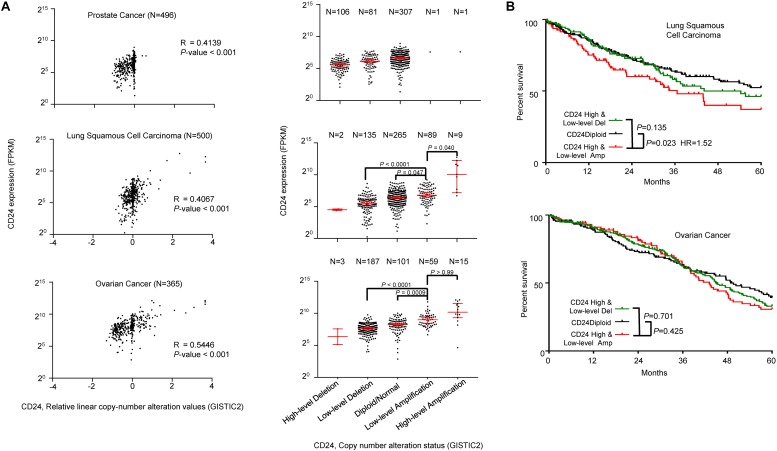
*CD24* amplification in different cancer types. **(A)** Scatter plot (left) and dot plot (right) showing the positive correlation between *CD24* copy number values defined by GISTIC2 approach and mRNA expression values quantified by FPKM in prostate cancer (top), lung squamous cell carcinoma (middle) and ovarian cancer (bottom). **(B)** Kaplan–Meier survival curve comparing the OS time for different *CD24* copy number subtypes in lung squamous cell carcinoma (top) and ovarian cancer (bottom). The Spearman coefficient of correlation (R) and significance in panel **(A)** (left) was determined by linear regression. Statistical significance was determined by the One-way ANOVA in panel **(A)** (right) and log-rank test in panel **(B)**.

To determine the general significance of *CD24* amplification and cancer prognosis, we also analyzed the correlation between *CD24* gene copy number and mRNA expression among prostate, lung and ovarian cancer, all known to overexpress *CD24* (Fang et al., 2010). As shown in Figure 6A, *CD24* amplification was observed at a high rate among ovarian cancer (20.3%) and lung cancer (19.6%), while prostate cancer rarely (0.4%) showed *CD24* amplification. Nevertheless, a positive correlation was observed between gene copy number and *CD24* mRNA levels. Besides, a strong association was observed between *CD24* amplification and OS of lung cancer patients (Figure 6B). Surprisingly, despite a high rate of *CD24* amplification in ovarian cancer, such amplification has no prognostic value for OS rate (Figure 6B).

## Discussion

Numerous studies have shown *CD24* overexpression and its prognostic significance in multiple cancer types (Kristiansen et al., 2003; Fang et al., 2010). However, the cause of *CD24* overexpression remained largely enigmatic. Our analysis presented herein established a strong correlation between *CD24* overexpression and copy number amplification, thus suggested gene amplification as a potential mechanism for *CD24* overexpression. *CD24* plays several critical roles in cancer pathogenesis and cell surface *CD24* has long been associated with cancer metastasis through its role as selectin ligand (Aigner et al., 1997). Consistently, our data showed *CD24* copy number variation was correlated with expression levels of genes known for their association of cancer metastasis, including *CCL18, HDAC2*, and *DSC2*. *CD24* has oncogenic activity through its regulation of *Src*/*STAT3* pathway (Bretz et al., 2012). We have reported that *CD24* over-expression is critically important for the inactivation of mutant p53 protein in cancer cells (Wang et al., 2015). It followed that cancer samples with most *TP53* mutations must overexpress *CD24*. By showing a 2.6-fold enrichment of *TP53* mutations among *CD24* amplified samples, the data presented here provided further clinical support for the interaction between *CD24* and *TP53* (Wang et al., 2015; Li et al., 2018). The high rate of *CD24* amplification among multiple cancer types satisfies major criteria of *CD24* as a *bona fide* oncogene (Hanahan and Weinberg, 2011). Obviously, while gene amplification provides a genetic fix that facilitates *CD24* overexpression in the cancer cell, this is not the only mechanism by which *CD24* is induced at high levels among cancer. Only 2/496 prostate cancer patients show *CD24* amplification. Therefore, non-amplification mechanisms, such as activation of HIF1α (Thomas et al., 2012), may work either independently or in concert with gene amplification to drive a high level of *CD24* expression in cancer.

By comparing the OS of patients with or without *CD24* amplification, we showed that *CD24* amplification was among the most significant genetic prognostic indicator of OS for BRCA patients. Based onHR, the impact of *CD24* amplification (*HR* = 1.79) was considerably stronger than *TP53* mutations (*HR* = 1.35) and basal-like cancer type (*HR* = 1.32). Since the association remains robust and significant in multivariate analysis, *CD24* amplification can be considered as an independent diagnosis marker for BRCA patient prognosis. Besides, *CD24* amplification is enriched in basal-like BRCA patients which has a poorer prognosis, it is of interest to consider whether *CD24* amplification is the driving factor for poor prognosis. We consider *CD24* gene amplification as the primary factor in prognosis as the impact is undiminished after multivariate analysis and its impact is higher than basal-like features. The significance of *CD24* amplification is further enhanced as the frequency of *CD24* amplification is relatively high among major cancer types, including breast (18.4%), ovarian (20.3%), and lung cancer (19.6%). Surprisingly, *CD24* amplification in ovarian cancer does not associate withOS. One potential interpretation may relate to the fact that only intracellular *CD24* appears to affect ovarian cancer survival (Kristiansen et al., 2002). Therefore, factors that control *CD24* processing may mask the impact of gene amplification.

To our knowledge, this is the first demonstration of *CD24* copy number amplification during carcinogenesis. Despite functional role for *CD24* in aspects of cancer development, there is no clinical evidence that *CD24* expression is significant enough for cancer (Fang et al., 2010; Kwon et al., 2015). Our analysis revealed, previously undescribed and marked the oncogenic role of *CD24* copy number amplification in the BRCA. It is certainly convinced that the oncogenic features associated with *CD24* amplification cancers could influence response to BRCA treatment strategies. Lacking independent dataset and experimental validation is a limitation to this work, however, given the remarkably *CD24* amplification associated with oncogenic and prognosis signatures, we believe that this phenomenon is generalizable. The future analysis of other datasets with large cancer patients cohort and abundant experimental results will be important to confirm these findings. An increasing amount of evidence demonstrates that non-coding RNAs (ncRNAs), particularly microRNAs (miRNAs) (Lu et al., 2005; Chen et al., 2019) and long non-coding RNAs (lncRNAs) (Huarte, 2015; Chen et al., 2017), are aberrantly expressed in several complex diseases, including various cancers. And many high-quality computational inquiries into the genomic investigation of ncRNA–gene-cancer associations (Chen and Yan, 2013; Chen and Huang, 2017; Chen et al., 2018a, b), revealed the prognosis value and drug target potential for clinical use. There may be many ncRNA-mediated epigenetic changes to *CD24* expression to be discovered in the future, as an important complement to overexpression and copy number amplification, provide a more accurate prediction for clinical outcome. Besides, the machine-based-learning approaches are being developed to aid the diagnosis of clinical samples. A variety of these machine learning techniques, including decision trees, Bayesian networks, support vector machines, and convolutional neural networks have been widely applied in cancer research for the development of predictive models, resulting in effective and accurate decision making (Kourou et al., 2015; Montazeri et al., 2016; Esteva et al., 2019; Li et al., 2019). We believe that an approach with integrated molecular feature analysis (like oncogenic copy number amplification analysis) and machine learning prediction models in the future will strengthen the capabilities of cancer diagnosis, prognosis, and even treatment.

Genotyping for *CD24* copy number variation could provide a simple selection or stratification factor to identify populations of interest for cancer risk and treatment subtype, and its exploration as a predictive biomarker is warranted. We believe the *CD24* amplification could serve as a promising therapeutic target and prognosis marker.

## Data Availability

Publicly available datasets were analyzed in this study. This data can be found here: https://portal.gdc.cancer.gov/.

## Author Contributions

PeZ performed the analysis and prepared the manuscript. YL and PaZ supervised the studies and revised the manuscript.

## Conflict of Interest Statement

YL and PaZ are co-founders of, and have equity interests in OncoImmune, Inc. The remaining author declares that the research was conducted in the absence of any commercial or financial relationships that could be construed as a potential conflict of interest.

## Footnotes

1 https://portal.gdc.cancer.gov/, Accessed on February 2018

2 http://www.cbioportal.org/index.do, Accessed on February 2018

3 ftp://ftp.broadinstitute.org/pub/GISTIC2.0/refgenes/

4 https://www.r-project.org/

5 http://www.cbioportal.org/

## Supplementary Material

FIGURE S1 Correlation between *CD24* copy number alterations and mRNA expression. **(A)** Change of *CD24* mRNA expression between all tumor samples (*N* = 1082) and normal samples (*N* = 112) from TCGA BRCA cancer studies. **(B)** Change of *CD45* mRNA expression between *CD24* higher expression groups (*CD24* exp high, *N* = 541) and *CD24* lower expression samples (*CD24* exp low, *N* = 541) from TCGA BRCA studies. **(C)** Kaplan–Meier OS curves comparing the high and low expression value of *CD24* for two independent BRCA patient cohorts. **(D)** Kaplan–Meier metastasis survival curves comparing the high and low expression value of *CD24* for two independent BRCA patient cohorts. Statistical significance was determined by One-way ANOVA in panel **(A)**, the Wilcoxon test in panel **(B)**, and the log-rank test in panels **(C,D)**.

## Abbreviations

BRCA: breast cancer
ER: estrogen receptor
GSEA: gene-set enrichment analysis
HR: hazard ratio
IHC: immunohistochemistry
OS: overall survival
PR: progesterone receptor
TCGA: the cancer genome atlas
TNBC: triple-negative breast cancers.

## References

[B1] Aigner S. Sthoeger Z. M. Fogel M. Weber E. Zarn J. Ruppert M. (1997). CD24, a mucin-type glycoprotein, is a ligand for p-selectin on human tumor cells. *Blood* 89 3385–3395. 10.1063/1.4773993 9129046

[B2] Badve S. Dabbs D. J. Schnitt S. J. Baehner F. L. Decker T. Eusebi V. (2011). Basal-like and triple-negative breast cancers: a critical review with an emphasis on the implications for pathologists and oncologists. *Mod. Pathol.* 24 157–167. 10.1038/modpathol.2010.200 21076464

[B3] Bartlett J. M. S. Going J. J. Mallon E. A. Watters A. D. Reeves J. R. Stanton P. (2001). Evaluating HER2 amplification and overexpression in breast cancer. *J. Pathol.* 195 422–428. 10.1002/path.971 11745673

[B4] Bretz N. P. Salnikov A. V. Perne C. Keller S. Wang X. Mierke C. T. (2012). CD24 controls Src/STAT3 activity in human tumors. *Cell. Mol. Life Sci.* 69 3863–3879. 10.1007/s00018-012-1055-9 22760497 PMC11114558

[B5] Castilla M. Á López-García M. Á Atienza M. R. Rosa-Rosa J. M. Diáz-Martín J. Pecero M. L. (2014). VGLL1 expression is associated with a triple-negative basal-like phenotype in breast cancer. *Endocr. Relat. Cancer* 21 587–599. 10.1530/ERC-13-0485 24891455

[B6] Chanrion M. Negre V. Fontaine H. Salvetat N. Bibeau F. Mac Grogan G. (2008). A gene expression signature that can predict the recurrence of tamoxifen-treated primary breast cancer. *Clin. Cancer Res.* 14 1744–1752. 10.1158/1078-0432.CCR-07-1833 18347175 PMC2912334

[B7] Cheang M. C. U. Voduc D. Bajdik C. Leung S. McKinney S. Chia S. K. (2008). Basal-like breast cancer defined by five biomarkers has superior prognostic value than triple-negative phenotype. *Clin. Cancer Res.* 14 1368–1376. 10.1158/1078-0432.CCR-07-1658 18316557

[B8] Chen J. Yao Y. Gong C. Yu F. Su S. Chen J. (2011). CCL18 from tumor-associated macrophages promotes breast cancer metastasis via PITPNM3. *Cancer Cell* 19 541–555. 10.1016/j.ccr.2011.02.006 21481794 PMC3107500

[B9] Chen J. C. Alvarez M. J. Talos F. Dhruv H. Rieckhof G. E. Iyer A. (2016). Erratum: identification of causal genetic drivers of human disease through systems-level analysis of regulatory networks. *Cell* 166:1055. 10.1016/j.cell.2016.07.036 27518566

[B10] Chen X. Huang L. (2017). LRSSLMDA: laplacian regularized sparse subspace learning for MiRNA-disease association prediction. *PLoS Comput. Biol.* 13:e1005912. 10.1371/journal.pcbi.1005912 29253885 PMC5749861

[B11] Chen X. Wang L. Qu J. Guan N.-N. Li J.-Q. (2018a). Predicting miRNA–disease association based on inductive matrix completion. *Bioinformatics* 34 4256–4265. 10.1093/bioinformatics/bty503 29939227

[B12] Chen X. Yin J. Qu J. Huang L. (2018b). MDHGI: matrix decomposition and heterogeneous graph inference for miRNA-disease association prediction. *PLoS Comput. Biol.* 14:e1006418. 10.1371/journal.pcbi.1006418 30142158 PMC6126877

[B13] Chen X. Xie D. Zhao Q. You Z.-H. (2019). MicroRNAs and complex diseases: from experimental results to computational models. *Brief. Bioinform.* 20 515–539. 10.1093/bib/bbx130 29045685

[B14] Chen X. Yan C. C. Zhang X. You Z.-H. (2017). Long non-coding RNAs and complex diseases: from experimental results to computational models. *Brief. Bioinform.* 18 558–576. 10.1093/bib/bbw060 27345524 PMC5862301

[B15] Chen X. Yan G.-Y. (2013). Novel human lncRNA–disease association inference based on lncRNA expression profiles. *Bioinformatics* 29 2617–2624. 10.1093/bioinformatics/btt426 24002109

[B16] Clarke C. Madden S. F. Doolan P. Aherne S. T. Joyce H. O’Driscoll L. (2013). Correlating transcriptional networks to breast cancer survival: a large-scale coexpression analysis. *Carcinogenesis* 34 2300–2308. 10.1093/carcin/bgt208 23740839

[B17] Desmedt C. Piette F. Loi S. Wang Y. Lallemand F. Haibe-Kains B. (2007). Strong time dependence of the 76-gene prognostic signature for node-negative breast cancer patients in the TRANSBIG multicenter independent validation series. *Clin. Cancer Res.* 13 3207–3214. 10.1158/1078-0432.ccr-06-2765 17545524

[B18] Esteva A. Robicquet A. Ramsundar B. Kuleshov V. DePristo M. Chou K. (2019). A guide to deep learning in healthcare. *Nat. Med.* 25 24–29. 10.1038/s41591-018-0316-z 30617335

[B19] Fang X. Zheng P. Tang J. Liu Y. (2010). CD24: from A to Z. *Cell. Mol. Immunol.* 7 100–103. 10.1038/cmi.2009.119 20154703 PMC4001892

[B20] Fillmore C. Kuperwasser C. (2007). Human breast cancer stem cell markers CD44 and CD24: enriching for cells with functional properties in mice or in man? *Breast Cancer Res.* 3 9–11. 10.1186/bcr1673 17540049 PMC1929090

[B21] Hanahan D. Weinberg R. A. (2011). Hallmarks of cancer: the next generation. *Cell* 144 646–674. 10.1016/j.cell.2011.02.013 21376230

[B22] Higuchi I. Kawai H. Kawajiri M. Fukunaga H. Horikiri T. Niiyama T. (1999). Statistically significant differences in the number of CD24 positive muscle fibers and satellite cells between sarcoglycanopathy and age-matched becker muscular dystrophy patients. *Intern. Med.* 38 412–415. 10.2169/internalmedicine.38.412 10397078

[B23] Hodis E. Watson I. R. Kryukov G. V. Arold S. T. Imielinski M. Theurillat J. P. (2012). A landscape of driver mutations in melanoma. *Cell* 150 251–263. 10.1016/j.cell.2012.06.024 22817889 PMC3600117

[B24] Hough M. R. Rosten P. M. Sexton T. L. Kay R. Humphries R. K. (1994). Mapping of CD24 and homologous sequences to multiple chromosomal loci. *Genomics* 22 154–161. 10.1006/geno.1994.1356 7959762

[B25] Huarte M. (2015). The emerging role of lncRNAs in cancer. *Nat. Med.* 21 1253–1261. 10.1038/nm.3981 26540387

[B26] Israel E. Kapelushnik J. Yermiahu T. Levi I. Yaniv I. Shpilberg O. (2005). Expression of CD24 on CD19-CD79a+early B-cell progenitors in human bone marrow. *Cell. Immunol.* 236 171–178. 10.1016/j.cellimm.2005.08.026 16181617

[B27] Kourou K. Exarchos T. P. Exarchos K. P. Karamouzis M. V. Fotiadis D. I. (2015). Machine learning applications in cancer prognosis and prediction. *Comput. Struct. Biotechnol. J.* 13 8–17. 10.1016/j.csbj.2014.11.005 25750696 PMC4348437

[B28] Kristiansen G. Denkert C. Dahl E. Pilarsky C. Hauptmann S. (2002). CD24 is expressed in ovarian cancer and is a new independent prognostic marker of patient survival. *Am. J. Pathol.* 161 1215–1221. 10.1016/S0002-9440(10)64398-2 12368195 PMC1867310

[B29] Kristiansen G. Winzer K.-J. Mayordomo E. Bellach J. Schlüns K. Denkert C. (2003). CD24 expression is a new prognostic marker in breast cancer. *Clin. Cancer Res.* 9 4906–4913. 14581365

[B30] Kwon M. J. Han J. Seo J. H. Song K. Jeong H. M. Choi J.-S. (2015). CD24 overexpression is associated with poor prognosis in luminal a and triple-negative breast cancer. *PLoS One* 10:e0139112. 10.1371/journal.pone.0139112 26444008 PMC4596701

[B31] Landemaine T. Jackson A. Bellahcène A. Rucci N. Sin S. Abad B. M. (2008). A six-gene signature predicting breast cancer lung metastasis. *Cancer Res.* 68 6092–6099. 10.1158/0008-5472.CAN-08-0436 18676831

[B32] Lawson D. A. Xin L. Lukacs R. U. Cheng D. Witte O. N. (2006). Isolation and functional characterization of murine prostate stem cells. *Proc. Natl. Acad. Sci. U.S.A.* 104 181–186. 10.1073/pnas.0609684104 17185413 PMC1716155

[B33] Lehmann B. D. Bauer J. A. Chen X. Sanders M. E. Chakravarthy A. B. Shyr Y. (2011). Identification of human triple-negative breast cancer subtypes and preclinical models for selection of targeted therapies. *J. Clin. Investig.* 121 2750–2767. 10.1172/JCI45014DS1 21633166 PMC3127435

[B34] Li D. Hu M. Liu Y. Ye P. Du P. Li C. S. (2018). CD24-p53 axis suppresses diethylnitrosamine-induced hepatocellular carcinogenesis by sustaining intrahepatic macrophages. *Cell Discov.* 4:6. 10.1038/s41421-017-0007-9 29423273 PMC5799181

[B35] Li O. Chang X. Zhang H. Kocak E. Ding C. Zheng P. (2006). Massive and destructive T cell response to homeostatic cue in CD24-deficient lymphopenic hosts. *J. Exp. Med.* 203 1713–1720. 10.1084/jem.20052293 16769998 PMC2118348

[B36] Li O. Zheng P. Liu Y. (2004). CD24 expression on T cells is required for optimal T cell proliferation in lymphopenic host. *J. Exp. Med.* 200 1083–1089. 10.1084/jem.20040779 15477346 PMC2211842

[B37] Li Y. Huang C. Ding L. Li Z. Pan Y. Gao X. (2019). Deep learning in bioinformatics: introduction, application, and perspective in the big data era. *arXiv* 10.1016/j.ymeth.2019.04.008 31022451

[B38] Liu Y. Zheng P. (2007). CD24: a genetic checkpoint in T cell homeostasis and autoimmune diseases. *Trends Immunol.* 28 315–320. 10.1016/j.it.2007.05.001 17531534

[B39] Lu J. Getz G. Miska E. A. Alvarez-Saavedra E. Lamb J. Peck D. (2005). MicroRNA expression profiles classify human cancers. *Nature* 435 834–838. 10.1038/nature03702 15944708

[B40] Mermel C. H. Schumacher S. E. Hill B. Meyerson M. L. Beroukhim R. Getz G. (2011). GISTIC2.0 facilitates sensitive and confident localization of the targets of focal somatic copy-number alteration in human cancers. *Genome Biol.* 12 1–14. 10.1186/gb-2011-12-4-r41 21527027 PMC3218867

[B41] Minn A. J. Gupta G. P. Padua D. Bos P. Nguyen D. X. Nuyten D. (2007). Lung metastasis genes couple breast tumor size and metastatic spread. *Proc. Natl. Acad. Sci. U.S.A.* 104 6740–6745. 10.1073/pnas.0701138104 17420468 PMC1871856

[B42] Montazeri M. Montazeri M. Montazeri M. Beigzadeh A. (2016). Machine learning models in breast cancer survival prediction. *Technol. Health Care* 24 31–42. 10.3233/THC-151071 26409558

[B43] Nielsen P. Cohen J. (1996). mCD24, a glycoprotein transiently inhibitor of neurite outgrowth expressed by neurons, is an inhibitor of neurite outgrowth. *Neuroscience* 16 2624–2634. 10.1523/jneurosci.16-08-02624.1996 8786438 PMC6578769

[B44] Ormandy C. J. Musgrove E. A. Hui R. Daly R. J. Sutherland R. L. (2003). Cyclin D1, EMS1 and 11q13 ampli cation in breast cancer. *Breast Cancer Res. Treat.* 78 323–335. 10.1023/A:1023033708204 12755491

[B45] Rakha E. A. Reis-Filho J. S. Ellis I. O. (2008). Basal-like breast cancer: a critical review. *J. Clin. Oncol.* 26 2568–2581. 10.1200/JCO.2007.13.1748 18487574

[B46] Ramaswamy V. Remke M. Bouffet E. Bailey S. Clifford S. C. Doz F. (2016). Risk stratification of childhood medulloblastoma in the molecular era: the current consensus. *Acta Neuropathol.* 131 821–831. 10.1007/s00401-016-1569-6 27040285 PMC4867119

[B47] Ray P. S. Wang J. Qu Y. Sim M. S. Shamonki J. Bagaria S. P. (2010). FOXC1 is a potential prognostic biomarker with functional significance in basal-like breast cancer. *Cancer Res.* 70 3870–3876. 10.1158/0008-5472.CAN-09-4120 20406990

[B48] Rodriguez-Pinilla S. M. Jones R. L. Lambros M. B. K. Arriola E. Savage K. James M. (2007). MYC amplification in breast cancer: a chromogenic in situ hybridisation study. *J. Clin. Pathol.* 60 1017–1023. 10.1136/jcp.2006.043869 17158641 PMC1972423

[B49] Roy S. S. Gonugunta V. K. Bandyopadhyay A. Rao M. K. Goodall G. J. Sun L. Z. (2014). Significance of PELP1/HDAC2/miR-200 regulatory network in EMT and metastasis of breast cancer. *Oncogene* 33 3707–3716. 10.1038/onc.2013.332 23975430 PMC3935988

[B50] Sagiv E. Starr A. Rozovski U. Khosravi R. Altevogt P. Wang T. (2008). Targeting CD24 for treatment of colorectal and pancreatic cancer by monoclonal antibodies or small interfering RNA. *Cancer Res.* 68 2803–2813. 18413748 10.1158/0008-5472.CAN-07-6463

[B51] Schuldenfrei A. Belton A. Kowalski J. Talbot C. C. Di Cello F. Poh W. (2011). HMGA1 drives stem cell, inflammatory pathway, and cell cycle progression genes during lymphoid tumorigenesis. *BMC Genomics* 12:549. 10.1186/1471-2164-12-549 22053823 PMC3245506

[B52] Shackleton M. Simpson K. J. Stingl J. Smyth G. K. Wu L. Lindeman G. J. (2006). Generation of a functional mammary gland from a single stem cell. *Nature* 439 84–88. 10.1038/nature04372 16397499

[B53] Sleeman K. E. Kendrick H. Ashworth A. Isacke C. M. Smalley M. J. (2006). CD24 staining of mouse mammary gland cells defines luminal epithelial, myoepithelial / basal and non-epithelial cells. *Breast Cancer Res.* 8 6–11. 10.1186/bcr1371 16417656 PMC1413978

[B54] Sorlie T. Tibshirani R. Parker J. Hastie T. Marron J. S. Nobel A. (2003). Repeated observation of breast tumor subtypes in independent gene expression data sets. *Proc. Natl. Acad. Sci. U.S.A.* 100 8418–8423. 10.1073/pnas.0932692100 12829800 PMC166244

[B55] Subramanian A. Tamayo P. Mootha V. K. Mukherjee S. Ebert B. L. Gillette M. A. (2005). Gene set enrichment analysis: a knowledge-based approach for interpreting genome-wide expression profiles. *Proc. Natl. Acad. Sci. U.S.A.* 102 15545–15550. 10.1073/pnas.0506580102 16199517 PMC1239896

[B56] Tang S. W. Chang W. H. Su Y. C. Chen Y. C. Lai Y. H. Wu P. T. (2009). MYC pathway is activated in clear cell renal cell carcinoma and essential for proliferation of clear cell renal cell carcinoma cells. *Cancer Lett.* 273 35–43. 10.1016/j.canlet.2008.07.038 18809243

[B57] Thomas S. Harding M. A. Smith S. C. Overdevest J. B. Nitz M. D. Frierson H. F. (2012). CD24 is an effector of HIF-1-driven primary tumor growth and metastasis. *Cancer Res.* 72 5600–5612. 10.1158/0008-5472.CAN-11-3666 22926560 PMC3488144

[B58] Wang L. Liu R. Ye P. Wong C. Chen G.-Y. Zhou P. (2015). Intracellular CD24 disrupts the ARF–NPM interaction and enables mutational and viral oncogene-mediated p53 inactivation. *Nat. Commun.* 6:5909. 10.1038/ncomms6909 25600590 PMC4300525

[B59] Wu G. Xing M. Mambo E. Huang X. Liu J. Guo Z. (2005). Somatic mutation and gain of copy number of PIK3CA in human breast cancer. *Breast Cancer Res.* 7 609–616. 10.1186/bcr1262 16168105 PMC1242128

