# Supplementary figures and images for: Amplification of the CD24 Gene Is an Independent Predictor for Poor Prognosis of Breast Cancer
Source: Front Genet. 2019 Jun 12;10:560. doi: 10.3389/fgene.2019.00560 (PMC6581687; doi:10.3389/fgene.2019.00560)

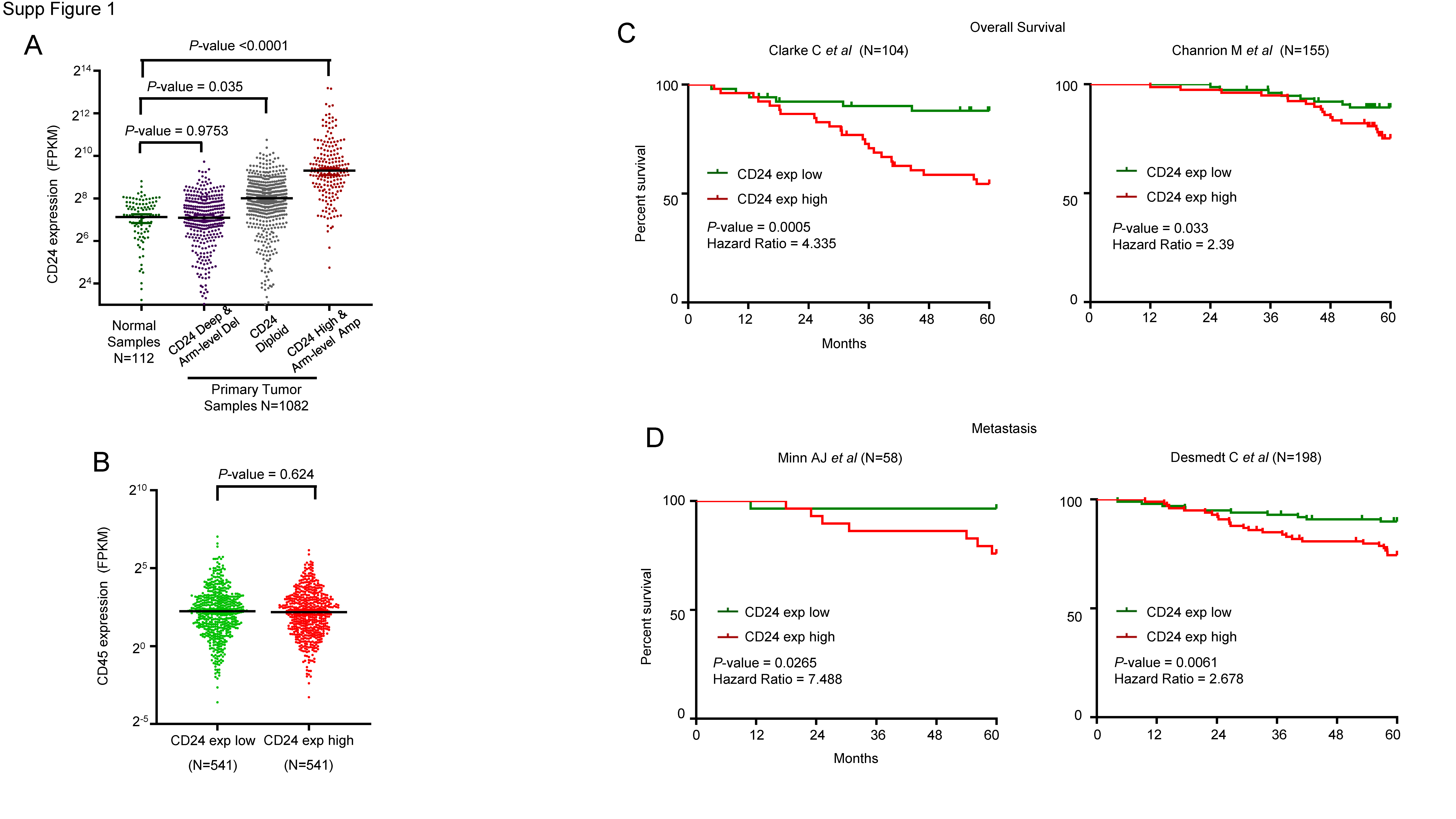

Supplement: FIGURE S1 — Correlation between CD24 copy number alterations and mRNA expression. (A) Change of CD24 mRNA expression between all tumor samples (N = 1082) and normal samples (N = 112) from TCGA BRCA cancer studies. (B) Change of CD45 mRNA expression between CD24 higher expression groups (CD24 exp high, N = 541) and CD24 lower expression samples (CD24 exp low, N = 541) from TCGA BRCA studies. (C) Kaplan–Meier OS curves comparing the high and low expression value of CD24 for two independent BRCA patient cohorts. (D) Kaplan–Meier metastasis survival curves comparing the high and low expression value of CD24 for two independent BRCA patient cohorts. Statistical significance was determined by One-way ANOVA in panel (A), the Wilcoxon test in panel (B), and the log-rank test in panels (C,D). [file Image_1.JPEG]
